# Effectiveness of Modified Flipped Classrooms Integrating Scenario-Based Questions, Multiple-Choice Question Assessments, and Mind Maps in Blood Physiology

**DOI:** 10.7759/cureus.81590

**Published:** 2025-04-01

**Authors:** Mayank Agarwal, Prabal Joshi

**Affiliations:** 1 Physiology, All India Institute of Medical Sciences, Raebareli, IND

**Keywords:** active learning, blood physiology, comprehension, medical education, scenario-based questions, students

## Abstract

Background

Conventional lectures in medical training may not always foster adequate comprehension and retention of complex physiological concepts. A modified flipped classroom integrating open-ended scenario-based questions (SBQs), multiple-choice questions (MCQs) assessments using an online response recording (ORR) system, and mind maps may enhance student comprehension, engagement, and knowledge retention. This study evaluates first-year Bachelor of Medicine and Bachelor of Surgery (MBBS) students' perceptions of an integrated instructional module on blood physiology.

Methods

This cross-sectional study was conducted at the All India Institute of Medical Sciences, Raebareli, India, from November 2024 to February 2025, involving 96 first-year MBBS students. The instructional module consisted of 21 structured lectures that utilized a modified flipped classroom approach with pre-class study materials and in-class PowerPoint-based teaching (Microsoft Corp., Redmond, WA). This approach was integrated with mind map navigations, SBQ discussions, and MCQ assessments via Google Forms (Google LLC, Mountain View, CA). Student perceptions were assessed using a validated 14-item questionnaire categorized into three domains: handout quality, comprehension and retention, and satisfaction and engagement. Additionally, two questions assessed the consistency of handout review before classes and the preferred method among SBQs, MCQs, and mind maps for enhancing understanding and retention. Responses were recorded on a five-point Likert scale. Data analysis included descriptive statistics, principal component factor analysis for construct validity, and Cronbach's alpha for reliability assessment.

Results

Students highly appreciated the quality of structured handouts (4.57 ± 0.42). Students believed that integrating SBQ discussions, MCQs with ORR, and mind maps helped improve knowledge retention and comprehension (4.28 ± 0.44) in a satisfactory and engaging environment (4.49 ± 0.41). Most students acknowledged the benefits of these methods: 91% agreed that MCQs reinforced key concepts, 96% reported an improved understanding through SBQ discussions, and 92% found mind maps helpful for knowledge retention. Additionally, 72% preferred the combined approach of SBQs, MCQs with ORR, and mind maps for comprehension and retention. However, only 26% consistently reviewed the handouts before classes.

Conclusion

The modified flipped classroom model integrating SBQs, MCQs with ORR, and mind maps helped students comprehend blood physiology while maintaining engagement in lectures. This structured instructional module offers a feasible and effective strategy for enhancing comprehension and engagement in medical education, even with limited resources.

## Introduction

A thorough understanding of blood physiology is essential for medical students, as it encompasses various complex processes vital for bodily function, including hemopoiesis, anemia, blood grouping and transfusion reactions, hemostasis, and immune responses [1]. Traditional didactic lectures, though valuable, may not always ensure optimal knowledge retention and engagement, potentially leading to gaps in comprehension [2].

The flipped classroom model reverses conventional teaching by requiring students to review provided study materials at home before engaging in interactive activities during in-class sessions [3]. However, the demanding academic schedule faced by Indian medical students, typically involving eight to nine hours of classes six days a week, can limit their capacity for extensive self-study [3]. An excessive academic workload has been linked to mental distress and adjustment disorders, conflicting with the "Three Eights" philosophy, which advocates an equal allocation of time for rest, work, and recreation [4-6].

A modified flipped classroom approach may offer a more practical solution to prevent cognitive overload. Providing study materials in advance and delivering didactic lectures on the same content can ensure proper learning opportunities. Students unable to review the material beforehand would still have the chance to grasp essential information and concepts during the lecture. This balanced approach would promote inclusivity and support all students in comprehensively understanding the subject.

Scenario-based short-answer question (SBQ) discussions can promote critical thinking, problem-solving skills, and knowledge retention [7,8]. Additionally, integrating multiple-choice questions (MCQs) with an online response recording (ORR) system can further strengthen active learning in large-group lectures [9]. Multiple-choice questions have become a widely accepted assessment tool in medical education [9]. However, limited research exists on student perceptions of screen-displayed MCQ-based assessments using Microsoft PowerPoint (Microsoft Corp., Redmond, WA) with a timer and responses recorded via Google Forms (Google LLC, Mountain View, CA), particularly as part of a sustainable, paper-free initiative. Another engaging technique involves using and discussing mind maps, which are diagrammatic representations that systematically organize information to enhance retention and conceptual clarity [10].

Despite their potential benefits, the integration of SBQs, MCQs with ORR, and mind maps in blood physiology lectures for first-year undergraduate medical students remains largely unexplored. This qualitative study aims to develop an instructional module that integrates these methods within a modified flipped classroom approach. Such an integrated module can enhance the learning experience by improving knowledge retention, comprehension, and problem-solving skills.

This study aims to evaluate first-year Bachelor of Medicine and Bachelor of Surgery (MBBS) students' perceptions of this integrated pedagogical approach, offering valuable insights into its effectiveness and potential for curriculum enhancement. This study is particularly relevant for Indian medical colleges, where resource constraints, limited adoption of innovative teaching methods, and insufficient technology integration challenge medical education [11-13].

## Materials and methods

Study design and setting

This cross-sectional study was conducted in the Department of Physiology at the All India Institute of Medical Sciences (AIIMS), Raebareli, Uttar Pradesh, India, between November 2024 and February 2025. Ethical clearance was obtained before the study began, and student participation was voluntary.

Participants and sample size

The study included first-year MBBS students from the 2024-2025 batch. The required sample size was calculated using Cochran's formula for proportion. It was determined that a minimum of 80 responses were necessary from a class of 100 to ensure that the margin of error did not exceed 5% at a 95% confidence interval.

Development of study tools

The first author developed the study materials for this project. A total of 21 lectures on blood physiology were created using Microsoft PowerPoint, following the Competency-Based Medical Education (CBME) framework established by the National Medical Commission (NMC) of India [1]. The lecture content was primarily based on the Guyton and Hall Textbook of Medical Physiology, Third South Asia Edition [14], with precautions taken to avoid plagiarism and ensure compliance with copyright laws.

The module employed a color-coded system where blue headings denoted essential must-know topics for professional examinations in the medical college. In contrast, orange headings represented may-know concepts relevant for future postgraduate entrance examinations.

Many original images and flowcharts were created using Microsoft PowerPoint and Whiteboard for Windows (Microsoft Corp.), with assistance from a drawing tablet to enhance visual learning. Twenty-five mind maps were developed using SimpleMind Pro trial version 2.4 for Windows (SimpleApps, Burlingame, CA). Each lecture featured an average of one mind map, although the number varied from none to three per lecture (Appendices A, B).

A total of 21 SBQs and 152 MCQs with four options and only one correct response were included in the module (Appendices C, D). Each lecture included an average of seven MCQs (ranging from five to nine) and one SBQ (ranging from 0 to two) to ensure an interactive and assessment-driven learning experience. A research committee composed of six faculty members from the Department of Physiology at AIIMS Raebareli, excluding the first author, validated these MCQs and SBQs.

A structured questionnaire was developed to assess student perceptions. It included 14 mandatory closed-ended items, with responses recorded on a five-point Likert scale (strongly agree, agree, neutral, disagree, strongly disagree). The questionnaire addressed three domains: (i) the quality of handouts (three items), (ii) comprehension and retention of the subject matter (five items), and (iii) satisfaction and engagement (six items). An additional item measured how often students reviewed the handouts before lectures, utilizing a five-point Likert scale (always, often, sometimes, rarely, never). A multiple-response question allowed students to indicate which method was the most effective in retaining and comprehending blood physiology content among MCQs, SBQs, and mind maps. Lastly, an optional open-ended question invited students to provide additional feedback. The departmental research committee validated the questionnaire.

Implementation strategy

Students were given a Google Drive (Google LLC) link to download lecture handouts in portable document format (PDF) at least a week before the scheduled class. These handouts contained concise, bulleted study material, mind maps, and SBQs. MCQs were intentionally excluded from the handouts.

Most of the one-hour lecture was organized into four segments. The first five to 10 minutes were allocated to reviewing the previous lecture through the navigation of the mind map, except for the first lecture, which cannot begin with mind map navigation. The mind map for the last lecture of the module was not discussed. This was followed by 25 to 30 minutes of PowerPoint-based lecture covering the content provided to students. Next, a 10 to 15-minute SBQs discussion session was conducted, which included open-ended, scenario-based, short-answer questions designed to reinforce the concepts. Finally, the last 10-15 minutes were for an MCQ assessment using ORR, accompanied by a brief discussion.

For the MCQ assessment, numbered questions with four choices were displayed on PowerPoint slides, with a one-minute timer per question. Students recorded their answers in Google Forms by selecting the correct option (a, b, c, or d) corresponding to each MCQ number. The Google Form contained only the question numbers with answer choices (a, b, c, d), without the actual MCQ text or options. A quick response (QR) code linking to the Google Form was generated using a free online tool and displayed on the screen before the assessment to facilitate easy access. Students who could not access the Google Form provided their responses on paper.

The blood physiology module lasted three months, from November 2024 to January 2025, and included 21 lectures as scheduled by the department. The first author took all the lectures. Students were informed that the marks obtained in the MCQs at the end of the lectures would not be included in the formative assessment. Following their first internal assessment in February 2025, students provided feedback through an anonymous questionnaire. Each item in the questionnaire was weighted equally, and the mean score for each category was calculated by averaging the Likert scale responses.

Statistical analysis

Online responses from all participants were compiled in Microsoft Excel 365 (Microsoft Corp). Descriptive statistical analysis was performed with IBM SPSS Statistics for Windows, version 27 (released 2020; IBM Corporation, Armonk, NY). Counts, percentages, and means with standard deviation (SD) were used to summarize the questionnaire responses. Principal component factor analysis of the questionnaire was conducted based on the following criteria: eigenvalues greater than one, varimax rotation with Kaiser normalization, and suppression of small coefficients with absolute values below 0.5. Internal consistency, or reliability, was assessed using Cronbach's alpha.

## Results

A total of 96 responses were obtained from a class of 100 students. Table 1 summarizes the responses to the 14 closed-ended questions across the three domains. The data show that students valued the quality of the handouts provided for the modified flipped classroom approach. They also indicated that the module helped them comprehend and retain concepts of blood physiology and increased their engagement in the classroom.

**Table 1:** Students' responses to the 14 closed-ended questions MCQ: multiple-choice question; SBQ: scenario-based question; Q: question

Domain and score (mean ± SD)	Question	Strongly agree (=5)	Agree (=4)	Neutral (=3)	Disagree (=2)	Strongly disagree (=1)	Mean ± SD
Quality of handouts (4.57 ± 0.42)	Q1. The provided lecture material was helpful for learning and revision	65 (68%)	30 (31%)	1(1%)	0	0	4.67 ± 0.50
	Q2. The PowerPoint presentations were clear and easy to understand	53 (55%)	41 (43%)	2 (2%)	0	0	4.53 ± 0.54
	Q3. The study material provided was comprehensive and sufficient for understanding blood physiology concepts	51 (53%)	44 (46%)	1 (1%)	0	0	4.52 ± 0.52
Comprehension and retention of subject matter (4.28 ± 0.44)	Q4. The MCQs at the end of each lecture helped to reinforce key concepts	27 (28%)	60 (63%)	9 (9%)	0	0	4.19 ± 0.59
	Q5. Scenario-based questions enhanced my overall understanding of blood physiology	42 (44%)	50 (52%)	4 (4%)	0	0	4.40 ± 0.57
	Q6. Navigating mind maps improved the overall retention of blood physiology	47 (49%)	41 (43%)	8 (8%)	0	0	4.41 ± 0.64
	Q7. I feel confident in recalling blood physiology concepts after completion of this module	24 (25%)	63 (66%)	9 (9%)	0	0	4.16 ± 0.57
	Q8. I feel confident in applying blood physiology concepts in clinical scenarios after completion of this module	29 (30%)	60 (63%)	7 (7%)	0	0	4.23 ± 0.57
Satisfaction and engagement (4.49 ± 0.41)	Q9. The integration of SBQs, MCQs, and mind maps made the lectures more engaging	48 (50%)	48 (50%)	0	0	0	4.50 ± 0.50
	Q10. The number of multiple-choice questions per lecture was adequate	53 (55%)	42 (44%)	1 (1%)	0	0	4.50 ± 0.52
	Q11. The time allocated per MCQ in the PowerPoint slides was sufficient for answering	36 (38%)	57 (59%)	3 (3%)	0	0	4.34 ± 0.54
	Q12. The overall format of the MCQ assessment (PowerPoint slides + Google Forms) was convenient and user-friendly	48 (50%)	46 (48%)	1 (1%)	1 (1%)	0	4.47 ± 0.58
	Q13. I am satisfied with the inclusion of SBQs, MCQs, and mind maps in the blood physiology lectures	48 (50%)	46 (48%)	2 (2%)	0	0	4.48 ± 0.54
	Q14. I would recommend incorporating SBQs, MCQs, and mind maps in lectures for future blood physiology students	57 (59%)	39 (41%)	0	0	0	4.59 ± 0.49

The Kaiser-Meyer-Olkin (KMO) test yielded a value of 0.849, confirming the sample's adequacy for factor analysis. Bartlett's test of sphericity was statistically significant (p < 0.001), indicating that the correlation matrix was suitable for factor extraction. The items were successfully loaded onto the three predetermined categories, as shown in Table 2. The overall questionnaire, including all 14 items, exhibited high internal consistency and reliability, with a Cronbach's alpha of 0.900.

**Table 2:** The factor loadings and communalities based on a principal components analysis; Cronbach's alpha for each domain indicated good reliability. Q: question

	Quality of handouts	Comprehension and retention of subject matter	Satisfaction and engagement	Communality
Q1	0.753			0.686
Q2	0.720			0.689
Q3	0.748			0.723
Q4		0.625		0.448
Q5		0.625		0.604
Q6		0.606		0.506
Q7		0.813		0.678
Q8		0.748		0.660
Q9			0.800	0.682
Q10			0.668	0.672
Q11			0.541	0.474
Q12			0.706	0.582
Q13			0.756	0.686
Q14			0.734	0.608
Cronbach's alpha	0.745	0.802	0.865	

**Figure 1:**
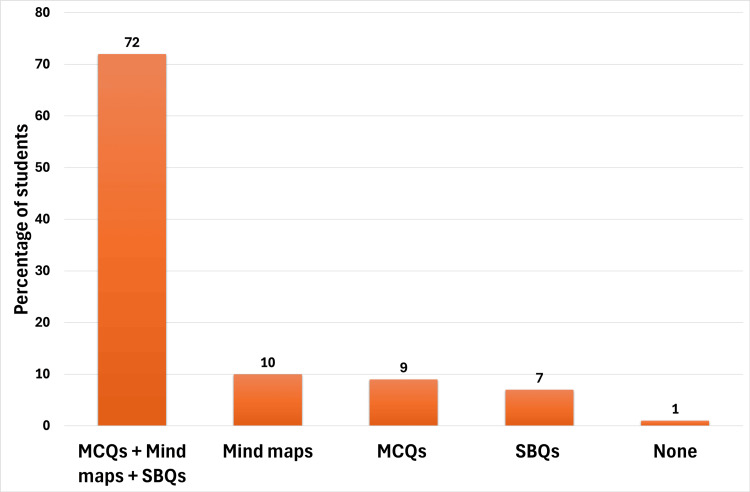
Student's response to "Which method was most helpful in understanding and retaining blood physiology concepts?" They had to choose all that apply: MCQs, mind maps, SBQs, and none. MCQ: multiple-choice question; SBQ: scenario-based question

Figure 1 illustrates students' perceptions of the effectiveness of three instructional methods, which were MCQs, SBQs, and mind maps, in understanding and retaining blood physiology concepts. The majority (69 students) found all three methods beneficial. Among the remaining students, 10 supported only mind maps, nine preferred only MCQs, and seven found that only SBQs were effective. One student indicated that none of the methods were helpful.

**Figure 2:**
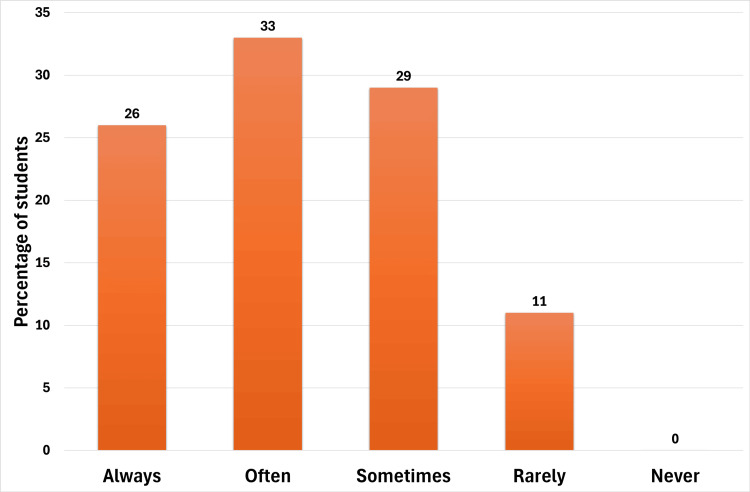
Student's response to "Did you review the handouts before lectures?" The students had to choose from always, often, sometimes, rarely, and never.

Figure 2 shows how often students reviewed handouts before lectures. Among the participants, 25 students reported that they reviewed the handouts consistently, 32 did so often, 28 sometimes, and 11 rarely.

Open-ended feedback highlighted areas for improvement. Some students suggested a slower lecture pace to enhance understanding. A few students recommended conducting SBQ and MCQ discussions in the subsequent class rather than at the end of lectures, allowing time for topic revision at home. Additionally, some students proposed increasing the number of MCQs and SBQs, particularly to aid their preparation for postgraduate entrance examinations.

## Discussion

This study explored the effectiveness of a modified flipped classroom that integrated SBQ discussions, MCQs with ORR, and mind maps in teaching blood physiology to first-year MBBS students. After their first internal assessment, students perceived that this integrated approach helped enhance their engagement, comprehension, and retention of blood physiology concepts.

Medical education in India faces unique challenges due to its rigorous curriculum, high student-to-faculty ratio, and reliance on traditional didactic teaching methods [11-13]. The NMC has emphasized the CBME curriculum to enhance students' critical thinking skills [1]. However, many institutions struggle to implement active learning strategies effectively due to resource constraints, faculty workload, and student resistance to self-directed learning [15]. This study addressed these challenges by developing an instructional module that integrated a modified flipped classroom approach with SBQs, MCQs with ORR, and mind maps. The findings highlight the feasibility and benefits of these methods, offering a sustainable framework for improving medical education in India.

Almost all students (98% to 99%) appreciated the quality of the handouts provided for the modified flipped classroom approach. Providing pre-class study material in a structured format gave students a foundation for in-class discussions, allowing them to focus on higher-order thinking skills during lectures. Our findings show that only 26% of students consistently reviewed the handouts before class, with the majority (62%) reporting that they reviewed them "often" or "sometimes." This finding highlights the necessity of a modified flipped classroom approach, where traditional didactic lectures complement pre-class material, ensuring inclusivity for students who could not complete pre-lecture preparation.

The high satisfaction and engagement scores suggest that students appreciated the integration of interactive methods, which have transformed the lectures into a student-centered learning experience. Most students (72%) acknowledged that combining MCQs, SBQs, and mind maps has helped enhance their understanding and knowledge retention. The overwhelmingly positive recommendation for incorporating these strategies into future lectures underscores the potential for broader implementation within the curriculum.

Incorporating recall-based and higher-order MCQs proved beneficial, as indicated by 91% of students who either agreed or strongly agreed that MCQs helped reinforce concepts in blood physiology. This finding aligns with existing literature suggesting that frequent MCQ-based assessments enhance knowledge retention and application [16,17]. The real-time response recording via Google Forms allowed immediate feedback, contributing to an efficient assessment process. Most students found the format convenient and the allocated response time sufficient. While most students found the number of MCQs per lecture adequate, some suggested increasing the number to better prepare for postgraduate entrance examinations. This result reflects a broader shift in medical education, where students increasingly value MCQs as learning tools and preparation strategies for high-stakes assessments.

Incorporating SBQ discussions into lectures provided students with a deeper understanding of blood physiology concepts, as acknowledged by 96% of participants. The open-ended feedback indicated that some students preferred a slower pace for SBQ discussions or their inclusion in the subsequent class to allow for better topic revision. This response suggests that while SBQ discussions were effective, their timing and integration into the lecture structure should be flexible to accommodate different learning preferences. Our study findings align with a recent study that suggests constructed-response short-answer questions promote deeper learning [8].

Mind maps were another key component of our instructional strategy. Approximately 92% of students found mind maps helpful in organizing information and reinforcing conceptual connections. These findings align with previous studies demonstrating that mind maps are effective cognitive tools for structuring and visualizing complex information, ultimately aiding long-term retention [10,18,19]. Our approach was to minimize the cognitive overload and ensure consistency in key concepts; thus, the instructor provided pre-made mind maps rather than traditional student-created maps. However, existing literature highlights that actively drawing mind maps may offer additional benefits regarding deeper processing and long-term retention [19]. Future studies could explore whether a blended approach, in which students are encouraged to develop their own mind maps using templates provided by instructors, enhances learning outcomes even further.

Strengths and limitations of the module

The module's structured format ensured a balanced approach, preventing cognitive overload while fostering an active learning environment. Research shows that students' attention spans during lectures decrease after about 20 minutes [20]. Therefore, limiting the lecture length to 25-30 minutes was a sensible approach. However, reducing the duration further was not practical due to the need to complete the syllabus within the required timeframe.

For the ORR-based MCQs, students logged in with their Google accounts. They entered their roll numbers into the Google Form, creating an automated attendance record that eliminated the need for manual attendance-taking, thereby saving time and effort. During MCQ assessments, the instructor verified student attendance by comparing the number of students in class with Google Forms records and the number of submitted papers, which were fewer than four per class.

Despite its strengths, the module had certain limitations. First, learning objectives for lectures were not displayed. For accurately constructed learning objectives, a department-wide consensus is necessary to standardize them. We were unable to draft learning objectives that included all faculty members for all 21 lectures. However, the study material was color-coded for 'must-know' and 'may-know' topics. Second, although SBQ sessions were incorporated, they lacked elaboration due to time constraints, limiting opportunities for in-depth discussions. Expanding these sessions in future iterations of the module could further enhance student engagement and comprehension. Third, all sessions were conducted by a single instructor, which limited direct interaction with each student compared to the more personalized engagement in small-group discussions. Fourth, the timings for the four lecture segments were not strictly consistent throughout the module, as the number of SBQs and MCQs varied depending on the topic's importance.

Limitations of the study

Despite the promising findings, this study has certain limitations. First, it was conducted at a single medical institution, which may limit the generalizability of the results to other settings. Second, while the study effectively measured student perceptions, objective learning outcomes such as exam performance were not assessed. The study relied on self-reported measures, which may introduce bias.

Future studies should incorporate longitudinal assessments of knowledge retention and academic performance to evaluate the long-term impact of this instructional module. Additionally, qualitative data from faculty perspectives could provide further insights into the feasibility and challenges of implementing such pedagogical innovations.

## Conclusions

The findings of this study support the integration of structured active learning strategies within a modified flipped classroom model. Students believed that the integration of SBQs, MCQs with ORR, and mind maps helps in classroom engagement, comprehension, and knowledge retention. Incorporating these instructional strategies could enable medical educators to create a compelling and engaging learning environment, effectively addressing the challenges of the demanding medical curriculum. Further research in diverse educational settings will help to generalize these findings and refine the module for broader implementation.

## Figures and Tables

**Figure 3:**
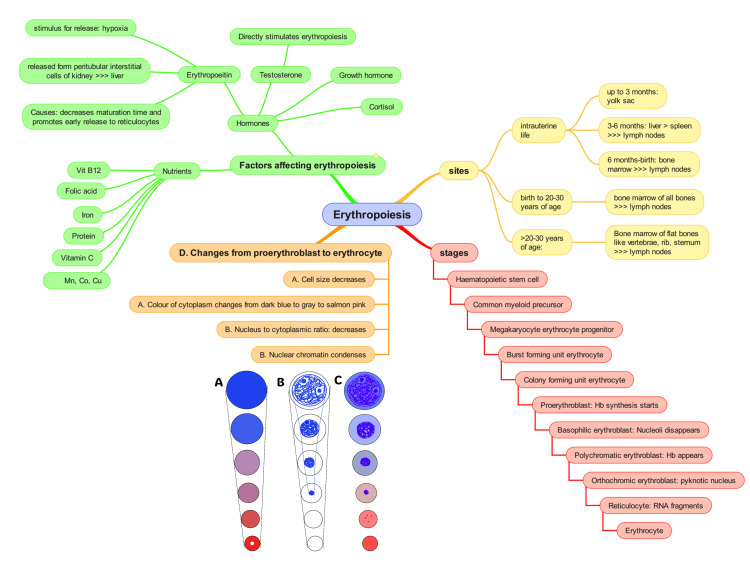
Mind map for erythropoiesis This is the original work of the first author created using the SimpleMind Pro trial version 2.4 for Windows.

**Figure 4:**
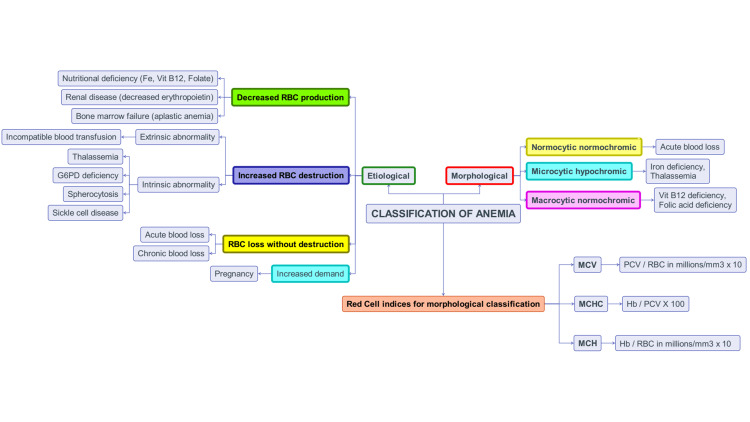
Mind map for morphological classification of anemia This is the original work of the first author created using the SimpleMind Pro trial version 2.4 for Windows.

**Figure 5:**
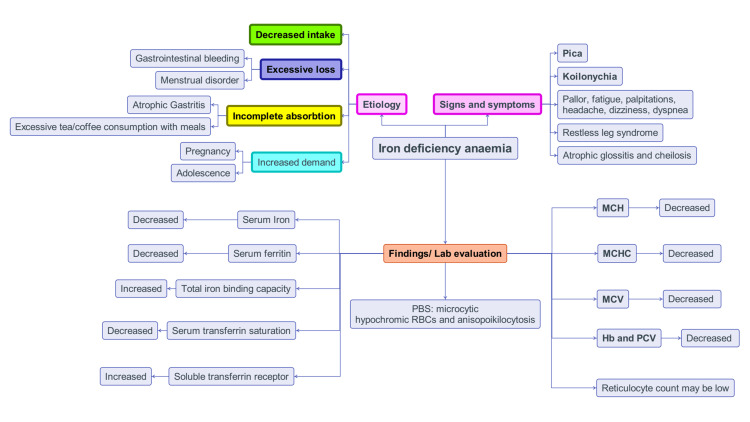
Mind map for iron deficiency anemia This is the original work of the first author created using the SimpleMind Pro trial version 2.4 for Windows.

**Figure 6:**
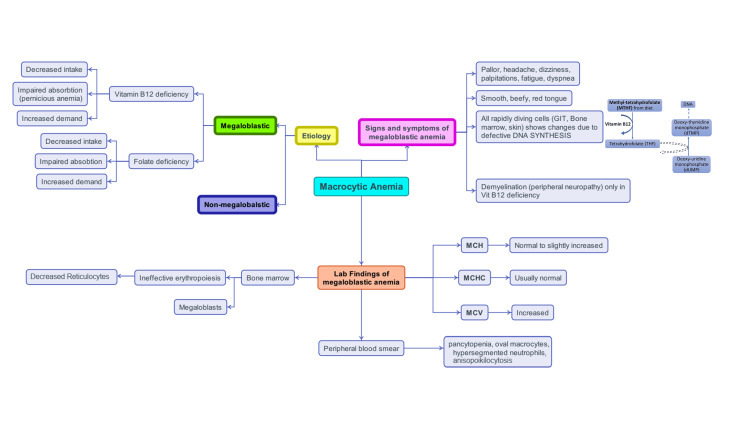
Mind map for macrocytic anemia This is the original work of the first author created using the SimpleMind Pro trial version 2.4 for Windows.

**Figure 7:**
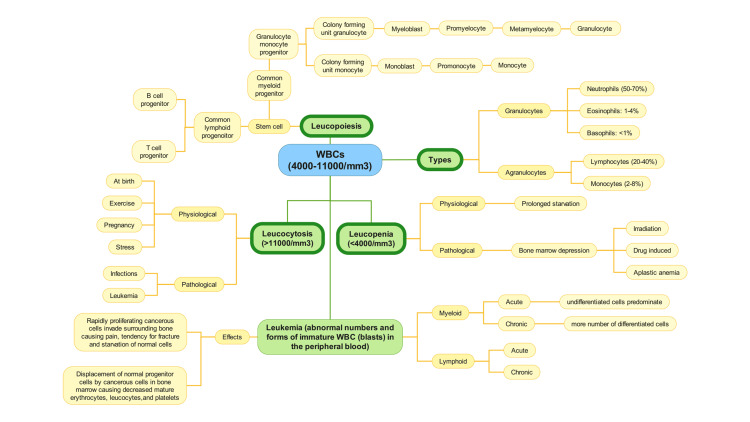
Mind map on leucocytes This is the original work of the first author created using the SimpleMind Pro trial version 2.4 for Windows.

**Figure 8:**
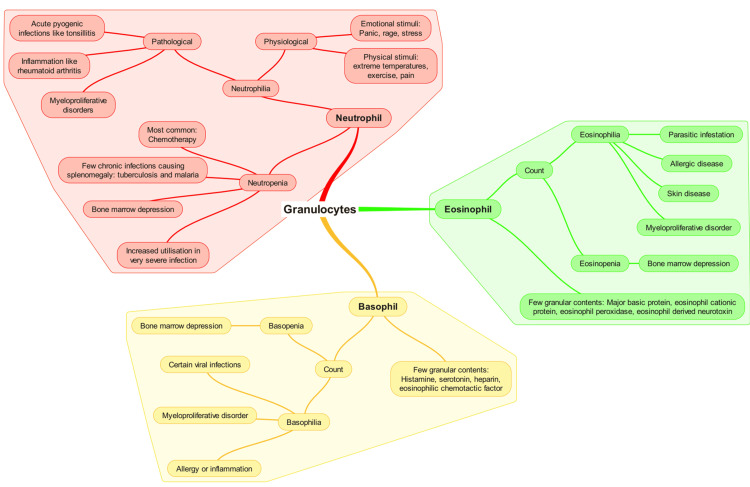
Mind map for few important causes of increase and decrease in granulocytes This is the original work of the first author created using the SimpleMind Pro trial version 2.4 for Windows.

**Figure 9:**
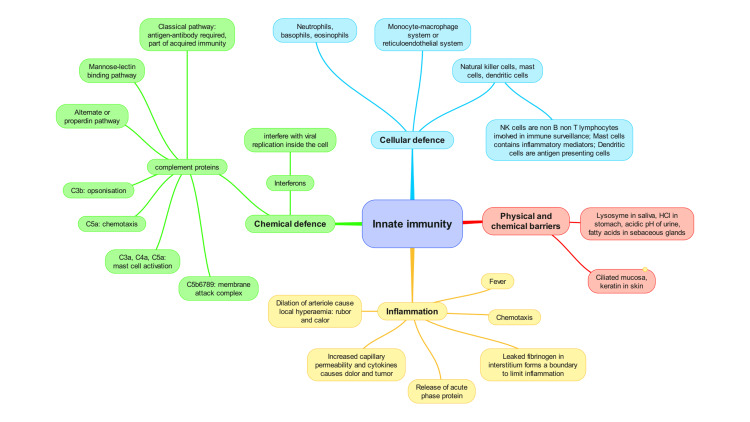
Mind map on innate immunity This is the original work of the first author created using the SimpleMind Pro trial version 2.4 for Windows.

**Figure 10:**
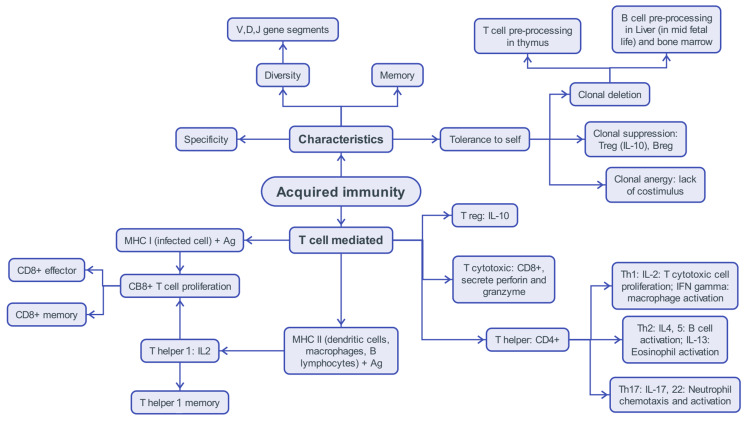
Mind map on acquired and cellular immunity This is the original work of the first author created using the SimpleMind Pro trial version 2.4 for Windows.

**Figure 11:**
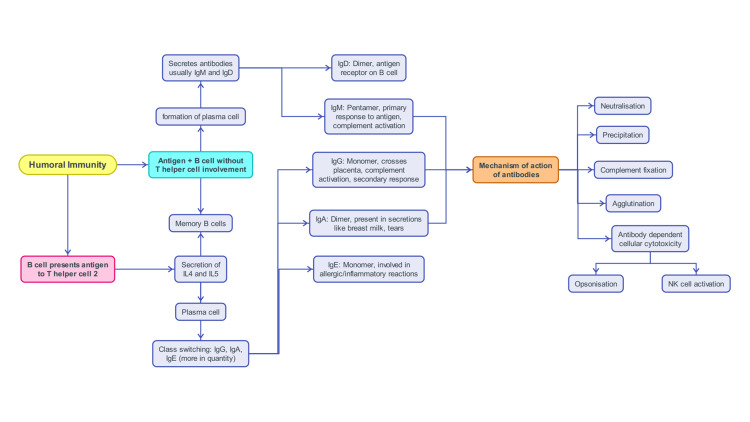
Mind map for humoral immunity This is the original work of the first author created using the SimpleMind Pro trial version 2.4 for Windows.

**Figure 12:**
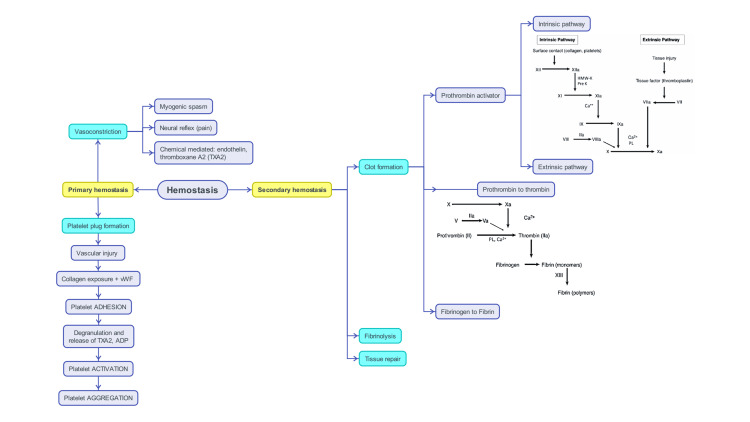
Mind map for hemostasis This is the original work of the first author created using the SimpleMind Pro trial version 2.4 for Windows.

**Figure 13:**
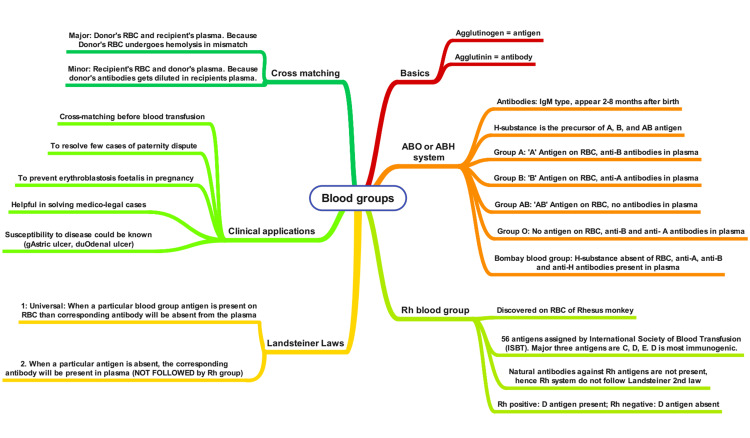
Mind map for blood groups This is the original work of the first author created using the SimpleMind Pro trial version 2.4 for Windows.

**Figure 14:**
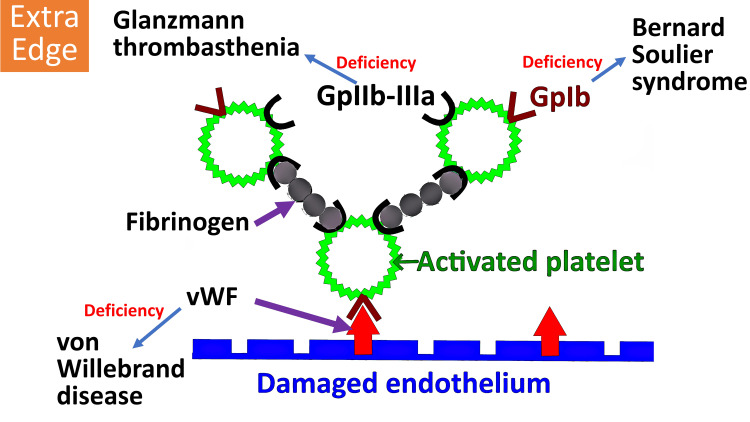
Platelet adhesion and aggregation defects This is the original work of the first author, created using Microsoft PowerPoint and Whiteboard for Windows.

**Figure 15:**
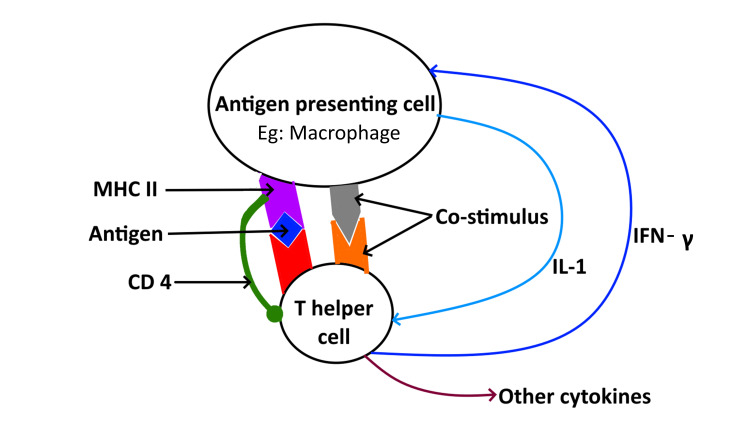
Activation of T helper cell by antigen presenting cell This is the original work of the first author, created using Microsoft PowerPoint and Whiteboard for Windows.

## Appendices

Appendix A

Mind Maps

A few mind maps are attached here for reference. These are the original works of the first author.

Appendix B

*Images Created for the Module*

These are original images created by the first author using Microsoft PowerPoint and Whiteboard.

Appendix C

Scenario-Based Questions

**Table 3:** Scenario-based questions

Scenario-based questions
1. A 46-year-old man presents with generalized oedema, shortness of breath, and fatigue. On examination, the patient has pitting oedema in both lower limbs and ascites. Laboratory investigations reveal low serum albumin, low total plasma protein, normal serum globulins, and high urinary protein. Based on these findings, the physician suspects nephrotic syndrome, which has led to significant protein loss through the urine. a. What is the primary physiological function of albumin, and how does its low level contribute to the patient's generalized oedema? b. Discuss the probable cause of normal serum globulin levels despite urinary protein loss in this case. c. Besides nephrotic syndrome, name two other conditions and mechanisms by which they can lead to hypoalbuminemia.
2. A 55-year-old man with chronic lung disease presents with persistent fatigue and shortness of breath due to impaired blood oxygenation. He was prescribed a portable oxygen tank with a nasal cannula to improve his oxygen levels. Before starting oxygen therapy, his red blood cell (RBC) count was 6.1 million/mm³. After using oxygen therapy for four months, his RBC count decreased to 5.2 million/mm³. a. Which hormone is responsible for the elevated RBC count before oxygen therapy? b. Explain the hormone's mechanism of action and its role in erythropoiesis. c. What physiological explanation accounts for the reduction in RBC count following four months of oxygen therapy in this patient?
3. A study compared regular blood donors to a matched group of non-donors, assessing haemoglobin, haematocrit, and serum ferritin levels over time. While non-donors maintained stable iron stores, donors experienced a gradual decline. Despite declining iron stores, donors did not develop anaemia, as indicated by normal haemoglobin and haematocrit values. Further, donors showed a heightened capacity for iron absorption compared to non-donors. a. Why do repeated blood donations result in lower iron stores? b. Explain how iron absorption increased in donors, mentioning the specific proteins and transporters involved in the absorption process. c. Which laboratory test provides information about the body's iron stores?
4. A 52-year-old chronic alcoholic male has complaints of yellowish discolouration of eyes, pain in the abdomen, and fatigue for the past 6 days. Laboratory investigation revealed serum bilirubin 3 mg/dL, conjugated bilirubin 1 mg/dL, unconjugated bilirubin 2 mg/dL, raised alanine transaminase and raised γ-glutamyl transferase. Based on these findings, answer the following questions: a. Why was yellow discolouration seen in the eyes first? b. Describe the pathophysiology of jaundice in this case and explain why total, direct, and indirect bilirubin were raised. c. What would be the level (increase or decrease) of urinary bilirubin in this case?
5. A 32-year-old female presents with complaints of severe pain in the abdomen, nausea, and vomiting. She also had complaints of fatigue, yellowing of the skin and eyes, clay-coloured, foul-smelling stool, dark-coloured urine, and intense itching that persisted for one week. On ultrasound, it was found that multiple stones obstructed the common bile duct. a. Explain why the patient's stool is clay-coloured and foul-smelling. b. Which liver enzymes would help in the diagnosis? c. Explain why urine is dark-coloured.
6. A 35-year-old woman developed fatigue and shortness of breath a few days after anti-malarial treatment, possibly due to glucose-6-phosphate dehydrogenase (G6PD) deficiency. She had yellow sclerae and dark-coloured urine. Lab investigation showed haemoglobin 10 g/dL, haematocrit 34%, 4 million/mm3 RBC count, normal liver enzyme levels, and fragmented RBCs in blood smear. a. Calculate the erythrocyte indices (MCH, MCV, MCHC). b. Explain the importance of peripheral blood smear findings in the diagnosis of anaemia. c. Explain why the patient had yellow-coloured eyes and dark urine.
7. A 5-year-old child from a poor slum dweller family is brought to the doctor with the complaint of often eating dirt. On examination, the patient had pallor, spoon-shaped nails, and an eroded angle of mouth. His haemoglobin concentration was 8 gm%. a. Discuss the probable cause of anaemia in this case. b. What would be the peripheral blood smear finding in this case? c. Discuss the laboratory tests that would confirm the diagnosis.
8. A 45-year-old woman presents with fatigue, breathlessness, and a tingling sensation in her hands and feet for the past few months. She had a pale palpebral conjunctiva and a smooth, sore tongue on examination. Blood investigations revealed a haemoglobin level of 9 g/dL, mean corpuscular volume (MCV) of 110 fL, and hyper-segmented neutrophils in the peripheral blood smear. a. What is the provisional diagnosis in the above case? b. How does the neurological involvement in this patient can be explained? c. Why did she have a smooth tongue?
9. A 16-year-old boy from Shimla involved in strenuous work is found to have a haemoglobin of 20 g/dL and an RBC count of 7 million/mm3. His total bilirubin level is normal, but erythropoietin is mildly increased. No abnormality was found during the systemic physical examination. a. What is the most probable cause of increased haemoglobin in this case? b. What could be suspected if the erythropoietin level had decreased and the remaining findings remained the same? c. Explain the mechanism of action of erythropoietin.
10. A 44-year-old woman reporting fatigue, shortness of breath on exertion, and general abdominal discomfort had a slightly yellowish sclera. Her haemoglobin, RBC count, and haptoglobin were low, while serum bilirubin, reticulocyte count, and lactate dehydrogenase (LDH) were raised. Red blood cell indices and a peripheral blood film examination were further ordered. a. What do low haptoglobin, high bilirubin, reticulocyte count, and LDH indicate? b. How could peripheral blood film examination help diagnose the case? c. Discuss the red cell indices and their importance in classifying anaemia.
11. A 24-year-old woman presents to the clinic with a two-day history of fever, swelling, redness, and tenderness around a small cut on her forearm sustained while gardening. Laboratory tests reveal a slight elevation in leucocytes count, particularly neutrophils. The patient has no history of chronic illness or immunodeficiency. a. Explain the physiological mechanisms by which neutrophils are recruited to the site of infection. b. Discuss the role of mast cells in amplifying the inflammatory response. c. Describe the inflammation process observed in the affected area and explain its significance in innate immunity.
12. A 65-year-old man with diabetes presents with a rapidly spreading infection on his left lower leg following a minor scrape. The area is swollen, red, warm, and painful, with noticeable pus and necrotic tissue. Laboratory tests indicate elevated C-reactive protein (CRP) levels and marked neutrophilia. The patient is diagnosed with cellulitis and treated with intravenous antibiotics. a. How does the innate immune system detect pathogens in the wound? b. Discuss the three pathways of complement activation and their relevance in clearing the infection. c. How does fever, as part of the innate immune response, enhance pathogen clearance?
13. A 10-year-old child develops a viral skin rash diagnosed as chickenpox. Despite the infection, her recovery is uneventful without complications. a. Describe how the cell-mediated immune response identifies and eliminates virus-infected cells. b. Discuss the physiological mechanism leading to lifelong immunity after recovery.
14. A 22-year-old college student visits the clinic with complaints of fatigue, mild persistent fever, night sweats, and cough with pain in the chest for 10 days. She had similar symptoms when she was 10 years old. On examination, the doctor notices swollen lymph nodes in her neck and suspects tuberculosis. Blood tests reveal mildly elevated levels of IgM but highly elevated IgG. a. Explain how B cells become activated and differentiate into plasma cells. b. Discuss the various mechanisms by which antibodies can provide immunity. c. Explain why IgG was raised more than IgM.
15. A 22-year-old medical student pricks her finger while handling a needle during a practical session. She notices a few drops of blood, but within minutes, the bleeding stops without any medical intervention. a. What physiological mechanisms and mediators contribute to the initial vasoconstriction following vascular injury? b. What are the key steps in platelet plug formation? c. How does endothelial function influence primary haemostasis?
16. A 48-year-old male undergoes surgery for a hip fracture. On the third postoperative day, he developed swelling and pain in his right leg and was diagnosed with deep vein thrombosis. While initiating anticoagulation therapy, the physician explains thrombin's dual role in coagulation and anticoagulation. a. Discuss the procoagulant and anticoagulant actions of thrombin. b. Discuss the anticoagulants that can be used in vitro and their mechanism of action.
17. A 26-year-old male presents with excessive bleeding from a minor cut sustained while shaving. He reports frequent nosebleeds and easy bruising over the past few months. Lab investigations revealed prolonged activated partial thromboplastin time (aPTT), while prothrombin time (PT), platelet count, and bleeding time were within normal range. a. How do the intrinsic and extrinsic coagulation pathways lead to fibrin clot formation? b. What factors could lead to an isolated prolongation of aPTT but a normal PT?
18. A 6-year-old boy is brought to the emergency department by his parents due to swelling and pain in both knees that occurred after a minor injury while the boy was playing football. His parents report that he frequently develops bruises and once had prolonged bleeding after a minor dental extraction. There is a maternal uncle with a history of frequent bleeding episodes. Lab investigations revealed normal platelet count, clot retraction time, bleeding time, prothrombin time (PT), but a prolonged activated partial thromboplastin time (aPTT). a. Discuss the differential diagnosis. What is the role of family history in differential diagnosis? b. Discuss the coagulation pathways to differentiate between PT and aPTT.
19. A 2-day-old male newborn is brought to the neonatal intensive care unit due to persistent oozing from the umbilical stump and bruising at injection sites. The mother had an uneventful pregnancy but declined vitamin K prophylaxis at birth. The infant has no signs of sepsis, and the mother did not take any anticoagulant medications during pregnancy. Lab investigations showed a prolonged activated partial thromboplastin time (aPTT) and prothrombin time (PT) while bleeding time (BT) and platelet count were normal. a. Why are both PT and aPTT prolonged in vitamin K deficiency? b. Does the anticoagulation mechanism get affected in vitamin K deficiency?
20. The following results were obtained in the laboratory for the blood group test. Assume that none of these subjects ever had any blood transfusion. Subject’s A blood does not react with anti-A and anti-D but agglutinates with anti-B. Subject’s B blood does not react with anti-A, anti-D, and anti-B. Subject’s C blood does not react with anti-B but agglutinates with anti-A and anti-D. Subject’s D blood agglutinates with anti-A, anti-B, and anti-D. a. Who has blood group 'A'? b. Which antigens could be present on the RBCs of subject D? c. Explain which among these four subjects could donate to others in an emergency. d. If the blood of subject D is transfused to others, then theoretically, which subject would show the most potent reaction? Explain the answer.
21. A 28-year-old woman is experiencing her first pregnancy. She has a Rh-negative blood type, but her full-term newborn has a Rh-positive blood type. Shortly after birth, the baby appears unusually pale and yellowish. The medical team notes that Mrs. X had previously received an emergency blood transfusion. However, the details of the transfusion history are unclear. a. Explain the probable cause of the pale and yellowish skin of the newborn. b. Given that this was Mrs. X's first pregnancy, how can we explain the severity of the baby's condition? What role might her earlier blood transfusion have played? c. What severe complications can occur to the baby in this case? d. Assuming that this baby was born pink and healthy. What measures should be taken to prevent Rh alloimmunization in the future?

 Appendix D

Multiple-Choice Questions

**Table 4:** Multiple-choice questions

Question	Option a	Option b	Option c	Option d	Correct option
A 33-year-old male presents with generalized oedema and a reversed albumin: globulin ratio. What is the most likely cause of his oedema?	Impaired liver synthesis of albumin	Increased capillary permeability	Increased globulin production by the liver	Lymphatic obstruction	a
Which of the following statements about human blood is true?	Deoxygenated blood is bluish coloured	Venous blood has a higher pH than arterial blood	RBC is responsible for half of the viscosity of blood	Blood is isotonic with pure distilled water	c
A 56-year-old man with low albumin levels presents with generalized oedema. What is the primary role of albumin in preventing oedema in the circulatory system?	It increases the interstitial hydrostatic pressure	It maintains the oncotic pressure within the blood vessels	It acts as a buffering agent in the blood	It regulates the pH of the blood	b
A patient's laboratory test shows elevated C-reactive protein (CRP) levels. Which of the following is the best classification of CRP?	Coagulation factor	Transport protein	Immunoglobulin	Acute-phase protein	d
A 51-year-old woman with myasthenia gravis is undergoing plasmapheresis as part of her treatment. Which of the following best describes the primary purpose of plasmapheresis in this condition?	Enhancing the production of immunoglobulins	Removal of circulating autoantibodies	Increasing the number of red blood cells	Replacement of clotting factors	b
A 55-year-old patient undergoing chemotherapy develops low serum albumin levels. Which of the following best explains the mechanism by which chemotherapy may affect albumin levels?	Impaired hepatic function	Increased renal clearance of albumin from healthy kidney	Genetic predisposition	Increased degradation of albumin by the immune system	a
A 30-year-old man requires a bone marrow biopsy for evaluation of a haematological disorder. Which of the following is the best site for obtaining active bone marrow in this adult patient?	Femur	Distal radius	Iliac crest	Tibia	c
All hematopoietic cells are derived from the common myeloid progenitors, EXCEPT:	Lymphocytes	Platelets	Eosinophils	Erythrocytes	a
Which morphological changes are typically observed during normal haematopoiesis as the cell matures?	Cell diameter gradually increases	Cytoplasm gradually becomes dark blue	Nuclear chromatin gradually condenses	Formation of prominent nucleoli occurs	c
Which of the following is the earliest erythroid precursor to be identified morphologically in a standard bone marrow smear?	Proerythroblast	Basophilic erythroblast	Colony-forming unit erythrocyte (CFU-E)	Burst-forming unit erythrocyte (BFU-E)	a
Which of the following growth factors, produced in the kidneys, is used to treat anaemia associated with kidney disease?	Erythropoietin (EPO)	Thrombopoietin (TPO)	Granulocyte colony-stimulating factor (G-CSF)	Interleukin-3 (IL-3)	a
A 61-year-old man with chronic anaemia due to renal failure was started on erythropoietin therapy. Which of the following best explains how erythropoietin increases red blood cell production?	Inducing apoptosis of erythroid progenitors	Stimulating iron absorption from the gastrointestinal tract	Reducing the oxygen-binding capacity of haemoglobin	Stimulating the early release of reticulocytes from the bone marrow	d
In hypoxia, increased oxygen delivery is facilitated by ………………… erythropoietin production by the kidneys, which is under a …………………… feedback homeostatic control.	Increased; negative	Decreased; negative	Increased; positive	Decreased; positive	a
Which of the following best describes the crucial role of vitamin B12 and folic acid in haematopoiesis?	They support the synthesis of porphyrins	They act as cofactors in the absorption of iron	They promote the differentiation of erythroid and myeloid cells	They facilitate the production of thymidine triphosphate	d
Which RBC enzyme facilitates carbon dioxide transport?	Glucose-6-phosphate dehydrogenase (G6PD)	NADPH reductase	Carbonic anhydrase	Methaemoglobin reductase	c
Which factor of Fick's Law of diffusion is increased by the biconcave shape of an erythrocyte?	Surface area	Concentration gradient	Membrane permeability	Membrane thickness	a
A normal erythrocyte passes through narrower fenestrations in the spleen without being haemolyzed due to the following:	Absence of cell organelles	Biconcave shape of RBCs	Highly elastic RBC membrane	All of the above	d
A deficiency in glucose-6-phosphate dehydrogenase (G6PD) can lead to haemolytic anaemia. Which of the following best explains the effect of this enzyme deficiency on red blood cells?	Reduced ATP production	Inability to synthesize haemoglobin	Decreased protection against oxidative damage	Increased RBC deformability	c
A patient is diagnosed with hereditary spherocytosis. Which of the following is the primary defect in the red blood cell membrane that leads to the spherical shape of RBCs?	Absence of glycosylphosphatidylinositol (GPI)	Deficiency of spectrin protein	Absence of glucose-6-phosphate dehydrogenase (G6PD) enzyme	Presence of abnormal iron metabolism	b
On examination of the blood smear of a liver disease patient, acanthocytes, stomatocytes, echinocytes, and target cells were found. This condition will be termed as:	Rouleaux formation	Anisocytosis	Poikilocytosis	Agglutination	c
What is the total number of oxygen atoms that a fully oxygenated haemoglobin molecule can carry?	Two	Four	Eight	Sixteen	c
A researcher is exploring the factors influencing oxygen release from haemoglobin. A male infant's blood sample shows reduced binding capacity to 2,3-bisphosphoglycerate. What is the probable haemoglobin composition in this sample?	α2 β2	α2 γ2	β2 γ2	α2 δ2	b
Which of the following haemoglobin is not found in the normal adult?	α2 β2	α2 γ2	α2 δ2	α2 ε2	d
Iron absorption is increased by:	Phytate	Tannate	Alkali	Ascorbic acid	d
Which of the following regulates iron transport from intestinal cells into the bloodstream?	Transferrin	Hephaestin	Divalent metal transporter 1	Hepcidin	d
What is the primary storage form of iron that is readily available for metabolic processes?	Haemosiderin	Ferritin	Transferrin	Haemoglobin	b
Which of the following statements about haemoglobin structure is correct?	Each globin chain contains a haem group with iron in the ferric state	2,3-bisphosphoglycerate increases the affinity of haemoglobin for oxygen	Foetal haemoglobin exhibits a diminished affinity for oxygen compared to adult haemoglobin	None of the above	d
Which of the following statements about haemoglobin synthesis is correct?	Haemoglobin in adults is primarily synthesized in the liver	Synthesis of haem occurs in mitochondria and cytoplasm	β globin chains are maximally synthesized during foetal development	All of the above	b
Which hormone secreted by developing erythroblasts in bone marrow regulates iron absorption and recycling?	Hepcidin	Ferroportin	Erythroferrone	Erythropoietin	c
A newborn is diagnosed with hydrops fetalis and passes away shortly after birth. Genetic testing reveals a complete deletion of all four alpha globin genes. What is the composition of the predominant haemoglobin in this neonate?	Bart Hb	HbA	HbF	HbH	a
HbA1c level in a normal healthy adult is:	7-8.5 %	6-7.5%	5-6.5%	4-5.5%	d
If in the beta chain of haemoglobin, valine replaces glutamic acid, then:	Oxy-haemoglobin crystallizes to distort the RBC shape	Deoxy-haemoglobin becomes less soluble and polymerizes	Haemoglobin denatures and precipitates as Heinz bodies	Oxidation of iron in haem occurs	b
A newborn having sickle cell disease typically does not manifest the symptoms till 3-6 months of age because:	RBC count, and hence, haemoglobin levels are higher in newborns	The immune system is not developed in newborns, and hence, haemolysis of sickled RBC does not occur	Newborns up to 3-6 months of age have higher levels of HbF	Newborns have higher 2,3 diphosphoglycerate levels	c
The fundamental principle underlying the screening for sickle cell disease through a test utilizing a reducing agent (sodium dithionite) is that HbS in a reduced (deoxygenated) state:	Readily forms HbM, causing a colour change	Precipitates as Heinz bodies	Form insoluble polymer	Has iron in the ferric state that can be detected easily	c
An individual was diagnosed with thalassemia, and it was determined on electrophoresis that he has a high content of HbH. What is the abnormality in this patient?	Deletion of three genes synthesizing alpha chain	Deletion of three genes synthesizing beta chain	Deletion of one gene synthesizing alpha chain	Deletion of one gene synthesizing beta chain	a
Beta thalassemia results in ineffective erythropoiesis and haemolytic anaemia due to:	Precipitation of excess beta chains in erythrocytes and their precursors	Precipitation of excess alpha chains in erythrocytes and their precursors	Bone marrow suppression and splenomegaly	Precipitation of haemoglobin to Heinz bodies	b
Which of the following best explains the physiological significance of elevated foetal haemoglobin (HbF) in the beta-thalassemia major?	HbF has a lower affinity for oxygen, improving oxygen delivery to tissues	HbF binds 2,3-bisphosphoglycerate more efficiently, enhancing oxygen unloading	HbF production compensates for the lack of HbA	HbF leads to increased oxygen affinity, causing tissue hypoxia	c
A 42-year-old male presents with yellowing of the sclera and skin, dark urine, and pale stools. Laboratory tests show a total bilirubin level of 15 mg/dL, primarily due to conjugated bilirubin, mildly elevated aspartate transaminase (AST) and alanine aminotransferase (ALT), and markedly elevated alkaline phosphatase (ALP). What is the most likely cause of his jaundice?	Haemolytic anaemia	Acute viral hepatitis	Biliary obstruction	All of the above	c
A 25-year-old woman developed yellow sclerae and fatigue two days after being discharged from the hospital, where she was treated for malaria. Her lab results show an elevated indirect (unconjugated) bilirubin but normal conjugated bilirubin, normal liver enzymes, and a reduced haemoglobin level. What is the most likely mechanism of her jaundice?	Impaired hepatic conjugation of bilirubin	Haemolysis of red blood cells	Biliary obstruction	Hepatocellular injury	b
Which protein bindings free intravascular haemoglobin, thereby preventing the renal excretion of haemoglobin and facilitating its degradation?	Haptoglobin	Hemopexin	Ferritin	Albumin	a
The type of jaundice associated with increased urobilinogen without bilirubinuria is:	Acholuric	Obstructive	Hepatic	None of the above	a
A 45-year-old woman presents with fatigue, jaundice, and right upper quadrant pain. She has a history of Hepatitis B infection. Laboratory results show increased clotting time, aspartate transaminase (AST), alanine aminotransferase (ALT), direct and indirect bilirubin, and low serum albumin levels. Which of the following best explains her condition?	Post-hepatic jaundice	Acholuric jaundice	Hepatic jaundice	Pre-hepatic jaundice	c
In response (adaptation) to anaemia, which of the following is most likely to be reduced?	Rate and depth of respiration	Heart rate and cardiac output	Oxygen affinity of haemoglobin	Erythropoietin level	c
The most common presentation of anaemia is:	Fatigue and breathlessness on exertion	Jaundice and dark urine	Fever with chills and rigors	Pain in abdomen due to splenomegaly	a
The following is NOT a characteristic of microcytic hypochromic anaemia:	Small red blood cells	Central pallor less than 1/3rd of cell	Reduced MCV and MCHC	Decreased haemoglobin synthesis	b
If lab investigation reveals MCV 85 fL, MCH 20 pg, and MCHC 24%, what would be the morphological (Wintrobe's) classification of anaemia?	Normocytic hypochromic	Microcytic hypochromic	Normocytic normochromic	Microcytic normochromic	c
In patients with microcytic hypochromic anaemia, the serum bilirubin results might be misleading as an indicator of erythrocyte destruction because:	Bilirubin accumulates in the blood due to liver failure	High rate of erythropoiesis	The low haemoglobin content of cells causes less bilirubin formation	Small cells easily pass through the spleen and are not destroyed	c
Which of the following is correct for iron deficiency anaemia:	↑ total iron binding capacity, ↑ serum ferritin, ↑ % transferrin saturation	↓ total iron binding capacity, ↓ serum ferritin, ↓ % transferrin saturation	↑ total iron binding capacity, ↓ serum ferritin, ↑ % transferrin saturation	↑ total iron binding capacity, ↓ serum ferritin, ↓ % transferrin saturation	d
Elevated levels of antibodies that block intrinsic factor required for cobalamin absorption would NOT result in one of the following:	Increase in neutrophils having more than six lobes	Increased reticulocytes in circulation	Increased mean corpuscular volume	Decreased serum cobalamin	b
Which protein is indirectly assessed by total iron binding capacity in iron deficiency anaemia patients?	Ferritin	Transferrin	Hemopexin	Haptoglobin	b
A 25-year-old woman presents with complaints of fatigue, shortness of breath, and pale skin for the past few months. Her medical history is significant for heavy menstrual bleeding. Her serum ferritin is low, while folate and vitamin B12 are within normal range. Which one of the following findings would be present in this patient?	↑ MCV, ↓ MCH, ↓ MCHC	↓ MCV, ↓ MCH, ↓ MCHC	↑ MCV, ↑ MCH, ↑ MCHC	↓ MCV, ↓ MCH, ↑ MCHC	b
A 26-year-old patient with an RBC count of 2 million/mm3, haematocrit 30%, haemoglobin 9.3 g/dL, and total serum bilirubin 0.8 mg/dL, what could be the most likely pathology in this patient?	Iron deficiency anaemia	Folic acid deficiency anaemia	Thalassemia	Sickle cell anaemia	b
Find the false statement regarding megaloblastic anaemia.	Hyper-segmented neutrophils are the earliest manifestation	Decreased reticulocyte count	Increased mean corpuscular volume	Increased mean corpuscular haemoglobin concentration	d
All of the following characteristics are seen in a megaloblast, EXCEPT:	Megaloblast are larger than normoblast	Cytoplasmic maturation of megaloblast lags behind nuclear maturation	Nuclei of megaloblast have more open sieve-like chromatin than normoblast	Megaloblast can haemolyze mildly increasing serum unconjugated bilirubin	b
A 63-year-old man presents with anaemia, fatigue, and neuropathy. Laboratory tests revealed elevated levels of homocysteine, methylmalonic acid, and erythropoietin. Which of the following conditions is most likely in this patient?	Folate deficiency	Cobalamin deficiency	Iron deficiency	Chronic kidney disease	b
A 26-year-old strict vegetarian man with a chronic intestinal disease underwent treatment that involved removing only the distal ileum, keeping the rest of his intestine intact. What type of anaemia could this patient potentially develop?	Macrocytic normochromic	Microcytic hypochromic	Normocytic hyperchromic	Normocytic hypochromic	a
The primary abnormality that characterizes polycythaemia vera is:	Impaired synthesis of erythropoietin	Uncontrolled proliferation of erythroid precursors	High levels of erythropoietin	Increased destruction of mature red blood cells	b
Based only on high haematocrit and low erythropoietin levels, what could be the most probable provisional diagnosis among the following:	A resident of high-altitude	Polycythaemia vera	Chronic hypoxia due to lung disease	Chronic kidney disease	b
A 44-year-old woman presents with a ruddy complex and has complaints of hypertension. She had swollen and painful veins in her legs. Imaging reveals a mass in the right kidney. Her blood oxygen saturation was 99%. Blood tests show high levels of haemoglobin, haematocrit, and erythropoietin. Which of the following is the most probable diagnosis among the following?	Polycythaemia rubra vera	Severe dehydration	Erythropoietin secreting tumour causing secondary polycythaemia	Chronic hypoxia due to lung disease	c
Lab investigations of a patient showed 7 g/dL haemoglobin, 25% haematocrit, 85 fL mean corpuscular volume, 0.6 mg/dL total bilirubin, low reticulocyte count, and a low erythropoietin level. What is the most likely diagnosis?	Chronic kidney disease	Chronic blood loss	Sickle cell anaemia	Pernicious anaemia	a
A 33-year-old woman recently treated for malaria visits the clinic complaining of fatigue and yellow eyes. Her lab investigations revealed a 3.5 million/mm3 RBC count, 9 g/dL haemoglobin, 86 fL mean corpuscular volume, and 10% reticulocytes. What is the most likely explanation for this presentation?	Aplastic anaemia	Glucose-6-phosphate dehydrogenase (G6PD) deficiency	Iron deficiency	Cobalamin deficiency	b
A patient presents with worsening fatigue and yellow sclera. The peripheral blood smear shows small, dense red cells without central pallor. Laboratory findings show 8 g/dL haemoglobin, 42% mean corpuscular haemoglobin concentration, 78 fL mean corpuscular volume, 3 mg/dL total bilirubin, and 15% reticulocytes. What could be a likely explanation among the following:	Hereditary spherocytosis	Thalassemia	Sickle cell anaemia	All of the above	a
A seven-year-old African boy presents with severe back and chest pain that started three hours ago after walking briskly through a high-altitude hilly area. He reports having similar episodes in the past few months during intense physical activities. Laboratory findings show 11 g/dL haemoglobin and 8% reticulocyte count. Which of the following diagnoses is most probable?	Sickle cell anaemia	Chronic kidney disease	Cobalamin deficiency	Both a and b	a
A 36-year-old female patient presents with a total leukocyte count of 15,000/mm3. Which of the following conditions is LEAST likely to cause this finding?	Strenuous exercise	Bacterial infection	Prolonged starvation	Short-term administration of corticosteroids	c
If an adult's total leukocyte count is 14,000/mm3 and a differential leukocyte count shows 80% neutrophils. This represents:	Relative neutrophilia	Absolute neutrophilia	Relative neutropenia	Absolute neutropenia	b
A 56-year-old man with a normal complete blood count three months ago now presents with fatigue, pallor, easy bruising, and leucocytosis of 45,000/mm³. A peripheral blood smear reveals that 80% of the circulating leukocytes are immature granulocytes. What is the likely diagnosis?	Acute myelocytic leukaemia	Acute lymphocytic leukaemia	Chronic myelocytic leukaemia	Chronic lymphocytic leukaemia	a
A 60-year-old man has had a persistently elevated white blood cell count of 25,000/mm³ for 18 months. A peripheral blood smear reveals that most leukocytes are mature-appearing cells with large, round nuclei, without granules, and scant cytoplasm. What is the most likely diagnosis?	Acute myelocytic leukaemia	Chronic myelocytic leukaemia	Acute lymphocytic leukaemia	Chronic lymphocytic leukaemia	d
A patient diagnosed with chronic lymphocytic leukaemia presents with anaemia. Which of the following pathophysiological mechanisms is most likely responsible for this patient's anaemia development?	Increased erythropoietin production	Leukemic cell infiltration of erythroid precursors	Impaired iron absorption	Premature destruction of red blood cells	b
A patient with suspected appendicitis has a total leucocyte count of 20,000/mm³, with a differential showing 75% neutrophils, including 15% band-form, 20% lymphocytes, 4% monocytes, and 1% eosinophils. All other parameters were within the reference interval. These results are most likely due to mobilization/release of neutrophils from:	Proliferating/mitotic bone marrow compartment	Storage/postmitotic bone marrow compartment	Marginated pool in vessels	Circulating pool into tissues	b
A 30-year-old man had an acute sinusitis. A sputum sample reveals bacterial infection. Which of the following leukocytes is most likely elevated in his sputum?	Eosinophil	Basophil	Neutrophil	Monocyte	c
Which of the following cell's granules predominantly contains the major-basic protein:	Macrophage	Eosinophil	Basophil	Neutrophil	b
A 43-year-old man presents with chronic diarrhoea and a stool sample positive for parasitic infection. Which of the following leukocyte types is most likely to be elevated in his peripheral blood smear?	Eosinophils	Neutrophils	Lymphocytes	Basophils	a
A 6-year-old boy with a history of recurrent infections is diagnosed with chronic granulomatous disease (CGD). The patient's leucocytes are unable to generate reactive oxygen species. Which of the following enzymes is most likely deficient in this condition?	Superoxide dismutase	Glucose-6-phosphate dehydrogenase	NADPH Oxidase	Catalase	c
Why can the absolute neutrophil count be unreliable in severe acute bacterial infections?	Neutrophils are mobilized to the site of infection, reducing circulating levels	Bone marrow mitotic pool reserves may become depleted	Bacterial infection suppresses neutrophil production	All of the above	a
A 25-year-old woman presents with recurrent bacterial infections. Laboratory tests reveal a leukocyte adhesion deficiency due to a defect in selectin molecules. In this case, which ability of neutrophils would be disrupted?	Phagocytosis	Diapedesis	Degranulation	Margination	d
A genetic defect leading to impaired production of granulocyte-macrophage colony-stimulating factor (GM-CSF) would most likely result in:	Monocytosis	Eosinophilia	Monocytopenia	Lymphocytopenia	c
A 24-year-old woman is stung by a bee and develops an immediate hypersensitivity reaction characterized by wheezing, hypotension, and hives. Which of the following leukocytes plays the most critical role in this reaction?	Neutrophils	Basophils	Monocytes	Eosinophils	b
A 25-year-old man sustains a deep laceration to his leg. During the immune response, leukocytes migrate across the endothelial layer into the surrounding tissues. What is the process of leukocyte migration across the endothelium called?	Margination	Rolling	Adhesion	Diapedesis	d
A 32-year-old man is diagnosed with a bacterial infection. Activation of the alternate complement pathway (most primitive) plays a role in the immune response. Which of the following is required to initiate this pathway?	Antigen-antibody complex	Mannose-binding lectin	C3 hydrolysis	Both b and c	c
A patient is diagnosed with a viral infection. Type I interferons (interferon-alpha and interferon-beta) are produced as part of the innate immune response. What is the primary role of these interferons?	Activating the complement system	Opsonization of pathogen	Inhibiting viral replication in infected cells	Recruiting macrophages to the infection site	c
A patient with a genetic deficiency in NADPH oxidase suffers from recurrent infections with catalase-positive organisms. Which innate immune mechanism is dysfunctional that best explains this condition?	Complement activation	Interferon-gamma production	Respiratory burst	TLR (toll-like receptors) signalling	c
During an acute phase response, which changes occur in plasma proteins?	Increased fibrinogen, decreased albumin	Increased C-reactive protein, decreased fibrinogen	Increased transferrin, decreased haptoglobin	Increased albumin, decreased transferrin	a
Choose the INCORRECT statement regarding natural killer (NK) cells:	They are T-lymphocytes	They do not require prior sensitization to kill target cells	They are capable of destroying cancer cells	They are involved in both acquired and innate immunity	a
A 7-year-old boy presents with recurrent infections caused by encapsulated bacteria. Laboratory studies reveal a defect in C3, a key component of the complement system. Which of the following processes is most likely impaired in this patient?	Activation of B cells	Opsonization of pathogens	Recruitment of cytotoxic T cells	Production of immunoglobulins	b
A 25-year-old woman presents with an acute allergic reaction following peanut ingestion. Her blood tests reveal elevated levels of inflammatory mediators such as histamine, bradykinin, and serotonin. Which cell type is most likely involved in this reaction?	Natural killer cells	Mast cells	Eosinophils	Macrophages	b
Which of the following complement proteins primarily triggers mast cell activation and chemotaxis of neutrophils?	C3a	C3b	C5a	C5b6789	c
A 25-year-old male is infected with Mycobacterium tuberculosis. His immune system initiates a robust cell-mediated response. Which of the following cells plays the central role in recognizing the antigen presented by macrophages and coordinating the immune response?	CD4+ T lymphocytes	CD8+ T lymphocytes	B lymphocytes	Natural killer cells	a
A 12-year-old boy presents with recurrent viral infections. Laboratory tests reveal a deficiency in MHC class I molecules on his cell surfaces. Which immune cell type would be least effective in this patient?	CD4+ T lymphocytes	CD8+ T lymphocytes	B lymphocytes	Natural killer cells	b
Which of the following is a primary characteristic of Th1 (T-helper) cells in cell-mediated immunity?	Promoting antibody production	Releasing interleukin-4 to induce B-lymphocyte class switching	Inhibiting the proliferation of T cells	Secreting interferon-γ to activate macrophages	d
Which antigen-presenting cell most potently stimulates a naive helper T lymphocyte:	Langerhans cells	Macrophages	Dendritic cells	B lymphocytes	c
Which cytokines are secreted by regulatory T lymphocytes (Tregs) to suppress the immune response?	Interleukin-2	Interleukin 4 and 5	Interleukin 10	Interferon gamma	c
Which of the following is essential for killing infected cells by cytotoxic T lymphocytes?	Antibodies	Granzyme and perforin	Interleukin 4 and 5	Histamine and serotonin	b
Which immune cell kills infected cells without requiring prior antigen exposure?	CD4+ T lymphocytes	CD8+ T lymphocytes	Natural killer (NK) cells	B lymphocytes	c
A 30-year-old woman receives live-attenuated (live virus rendered non-pathogenic) polio vaccine virus. Which of the following best describes the type of immunity she develops?	Passive artificial immunity	Passive natural immunity	Active natural immunity	Active artificial immunity	d
A 3-year-old boy is given an injection of pre-formed antibodies after exposure to hepatitis A. This is an example of which type of immunity?	Active natural immunity	Active artificial immunity	Passive artificial immunity	Passive natural immunity	c
A newborn receives protective antibodies against diphtheria from maternal breast milk. Which of the following immunoglobulin classes is primarily responsible for this passive immunity?	IgA	IgE	IgG	IgM	a
A healthy, full-term newborn receives protection from infections due to maternal antibodies transferred in utero. Which of the following immunoglobulin classes mediates this protection?	IgA	IgE	IgG	IgM	c
A patient is infected with a bacterial pathogen, and their humoral immune response leads to the activation of the complement system. Which of the following immunoglobulin classes is most effective at activating the complement cascade?	IgM	IgE	IgD	IgA	a
A 29-year-old woman is exposed to chickenpox but has no symptoms. She recalls receiving two doses of live-attenuated (live virus rendered non-pathogenic) varicella vaccine in childhood. Which of the following best describes the mechanism protecting her from infection?	Passive immunity from vaccine-induced antibodies	Memory B and T lymphocytes rapidly responding to the antigen	Continuous antibody production since vaccination	Suppression of viral replication by innate immunity	b
A 44-year-old man steps on a rusty nail and is administered tetanus immunoglobulin. Which of the following best describes the mechanism of this treatment?	Activation of naïve B lymphocytes to produce antibodies	Neutralization of the tetanus toxin by pre-formed antibodies	Activation of memory B lymphocytes to produce antibodies	Stimulation of T helper lymphocytes to enhance antibody production	b
A 22-year-old woman experiences sudden wheezing, dyspnoea, and hypotension after eating peanut butter at a restaurant. She is diagnosed with an anaphylactic reaction. Which physiological process is responsible for the rapid onset of symptoms?	Complement C3 activation by immune complexes	Degranulation of mast cells and basophils releasing histamine	Formation of antibodies leading to activation of phagocytes	Activation of CD8+ T lymphocytes inducing tissue destruction	b
A 32-year-old male exhibiting symptoms such as fever, weight loss, and persistent oral thrush was human immunodeficiency virus (HIV) positive. Which of the following is compromised in this patient?	Innate immunity	Cell-mediated immunity	Humoral immunity	All of the above	d
A 50-year-old male develops jaundice, diarrhoea, and a maculopapular rash after a bone marrow transplant. Tests reveal infiltration of donor T-lymphocytes in the liver and intestinal tissues. Which of the following is the most probable explanation?	Recipient T-lymphocytes attacked donor antigens	Donor was immunocompromised	Recipient was immunocompromised	Both a and c	c
A kidney transplant is performed between two genetically unrelated humans. The patient began to show signs of rejection one week later. Which graft type is involved, and what is the primary mechanism of rejection?	Allograft; type II hypersensitivity	Xenograft; type III hypersensitivity	Isograft; type IV hypersensitivity	None of the above	d
A 30-year-old male with kidney failure receives a transplant from his identical twin. He does not require immunosuppressive therapy and has no signs of rejection. What type of graft has been used?	Isograft	Allograft	Autograft	Xenograft	a
Platelets contain specialized granules that release factors crucial for haemostasis. Which of the following correctly pairs the granule type with its primary content?	Alpha granules – ADP	Dense granules – Fibrinogen	Alpha granules – von Willebrand factor	Dense granules – Platelet-derived growth factor	c
A 25-year-old male presents to the emergency department with a history of minor trauma after a fall from his bicycle. Which of the following is NOT a typical function of platelets in response to this injury?	Forming a temporary plug to stop bleeding	Releasing factors that promote blood clot formation	Assisting in the repair of the injured blood vessel	Initiates the breakdown of the blood clot (fibrinolysis)	d
A medical student is observing a peripheral blood smear under a microscope. They are learning to identify different blood cells. Which characteristics would NOT be expected when observing platelets on this smear?	The presence of a nucleus	Size of approximately 1 to 4 µm	Cytoplasm appearing light blue with fine red-purple granules	A discoid (plate-like) shape	a
A patient with liver cirrhosis is found to have thrombocytopenia. The reduced platelet count is most likely due to impaired production of which of the following?	Erythropoietin	Thrombopoietin	Platelet-derived growth factor	Interleukin-10	b
A haematologist is studying platelet production in the bone marrow. Which of the following is NOT a characteristic of mature megakaryocytes before platelet formation?	Megakaryocyte formation involves endomitosis	Megakaryocytes with ploidy level 8N-64N form platelets	A single megakaryocyte forms four platelets	Megakaryocytes undergo cell fragmentation to produce platelets	c
A haematology researcher is analysing platelet function under an electron microscope. He observes small, electron-dense granules in the cytoplasm. Which substance is NOT likely to be stored in these granules?	von Willebrand factor	Calcium	Serotonin	ADP	a
During primary haemostasis, platelets adhere to the exposed sub-endothelium of an injured blood vessel because of:	Fibrinogen secreted from alpha granules of platelets	von Willebrand factor secreted from dense granules of platelets	Exposed collagen	Prostacyclin (PGI2)	c
Which of the following is the EARLIEST haemostatic event following vascular trauma?	Fibrin clot formation	Platelet adhesion	Vasoconstriction	Platelet aggregation	c
A 45-year-old man is started on aspirin therapy for cardiovascular disease prevention. Which of the following best describes how aspirin inhibits platelet function during primary haemostasis?	Irreversibly inhibits cyclooxygenase-1, reducing thromboxane-A₂ production	Blocking ADP receptors, reducing platelet aggregation	Reversible inhibition of thrombin, reducing platelet activation	Inhibition of fibrinogen binding to glycoprotein IIb/IIIa	a
A researcher is studying the role of endothelium in haemostasis. She observes that endothelial cells produce a molecule that inhibits platelet aggregation and promotes vasodilation. Which of the following is most likely responsible for this effect?	Thromboxane A₂	Prostacyclin (PGI₂)	Endothelin	von Willebrand factor (vWF)	b
A researcher is studying platelet activation and aggregation in a laboratory model. She observes that a specific substance stimulates platelet shape change and glycoprotein IIb/IIIa receptor activation. Which of the following substances is most likely responsible for this effect?	Nitric oxide	Prostacyclin (PGI₂)	Fibrinogen	ADP	d
During primary haemostasis, platelets adhere to the exposed subendothelial collagen of an injured blood vessel. Which of the following primarily mediates this adhesion?	Fibrinogen	Glycoprotein Ib	Glycoprotein IIb/IIIa	Thromboxane A₂	b
A pharmacologist studies the effects of a novel antiplatelet drug that inhibits the release of dense granule contents. Which of the following processes would be most affected by this drug?	Platelet adhesion to collagen	Platelet activation and recruitment	Secretion of von Willebrand factor	Synthesis of thromboxane A₂	b
A 30-year-old woman with a history of heavy menstrual bleeding is diagnosed with von Willebrand disease. Which of the following best describes the role of the von Willebrand factor in primary haemostasis?	Platelet adhesion	Platelet aggregation	Platelet degranulation	Platelet activation	a
Which of the following best describes the function of serotonin in primary haemostasis?	Inhibits platelet aggregation	Induces vasoconstriction	Decreases thromboxane A₂ production	Both a and b	b
A 25-year-old male presents with excessive bleeding following a minor dental procedure. Laboratory tests reveal a prolonged activated partial thromboplastin time (aPTT) but a normal prothrombin time (PT) and platelet count. Which of the following coagulation factors is most likely deficient in this patient?	VII	VIII	X	Fibrinogen	b
A researcher isolates a plasma protein that inhibits thrombin and activated factor X. Its activity is significantly enhanced in the presence of heparin. Which of the following proteins is most likely being studied?	Protein C	Protein S	Antithrombin III	Thrombomodulin	c
Which of the following is the primary role of calcium ions in the coagulation cascade?	Cleavage of fibrinogen to fibrin	Activation of platelets	Stabilization of fibrin clot	Assembly of coagulation factor complexes on phospholipid surfaces	d
Both prothrombin time and activated partial thromboplastin time will NOT be increased in deficiency of which coagulation factor?	II	V	VIII	X	c
A patient has a prolonged aPTT (activated partial thromboplastin time) and a normal PT (prothrombin time). The aPTT was corrected on infusing plasma that lacked coagulation factor IX. Which coagulation factor deficiency is possible in this patient?	II	VII	VIII	IX	c
Which of the following is NOT an effective anticoagulant in vitro?	Heparin	Warfarin (coumarin derivative)	Ethylenediamine tetraacetic acid (EDTA)	Sodium citrate	b
A newborn had an umbilical stump bleeding and intracranial haemorrhage. Laboratory tests show normal prothrombin time, activated partial thromboplastin time, and bleeding time, but clot formation is weak and unstable. What is the most likely diagnosis?	Coagulation factor VIII deficiency	High molecular weight kininogen deficiency	Coagulation factor XIII deficiency	Thrombocytopenia	c
A 45-year-old male presents with deep vein thrombosis. Laboratory tests reveal a deficiency of antithrombin III. Which of the following coagulation factors will be least affected by this condition?	II	VII	IX	X	b
A 25-year-old man with recurrent deep vein thrombosis (DVT) is found to have a protein C deficiency. What is the primary function of protein C?	Converts fibrinogen to fibrin	Activates plasminogen	Inhibits factors Va and VIIIa	Binds calcium for clot stabilization	c
A patient with a severe decrease in coagulation factor X activity would likely have a normal:	Prothrombin time (PT)	Thrombin time (TT)	Activated partial thromboplastin time (aPTT)	Bleeding time (BT)	d
Which of the following coagulation tests is most sensitive to warfarin therapy?	Bleeding time (BT)	Prothrombin time (PT)	Activated partial thromboplastin time (aPTT)	Thrombin time (TT)	b
Which of the following clinical symptoms is NOT typical of coagulation factor deficiency?	Hemarthrosis	Petechiae	Prolonged bleeding	Deep tissue bleeding	b
A 55-year-old woman presents with severe sepsis. Laboratory findings reveal prolonged prothrombin time (PT) and activated partial thromboplastin time (aPTT), thrombocytopenia, and elevated D-dimer levels. Which of the following best describes the underlying pathophysiology?	Deficiency of Protein C and antithrombin III	Uncontrolled activation of coagulation and fibrinolytic systems	Platelet dysfunction	Deficiency of plasmin	b
Which of the following represents the most appropriate initial therapeutic intervention for a patient with massive pulmonary embolism?	Infusion of plasma with high levels of citrate	Oral anticoagulant warfarin	Oral low-dose aspirin	Injecting drug alteplase, which is a tissue plasminogen activator	d
A patient, after three days of initiation on warfarin therapy, had an INR of 0.7. How would you interpret this result?	The anticoagulation therapy is adequate for this patient	The patient is at risk of blood clot formation	The patient should immediately receive a vitamin K injection	The patient had coagulation factor VII deficiency	b
A 21-year-old male had hemarthrosis after a minor injury while playing football. Investigations revealed normal platelet count, clot retraction time, and prothrombin time (PT), but activated partial thromboplastin time (aPTT) and bleeding time (BT) were prolonged. What could be the probable diagnosis?	Haemophilia	Vitamin K deficiency	Thrombocytopenia	von Willebrand Disease	d
A 30-year-old woman develops excessive bleeding after a caesarean section. Her lab results show prolonged prothrombin time, activated partial thromboplastin time, and bleeding time. She had a low fibrinogen level and thrombocytopenia. Which of the following is the most likely diagnosis?	Haemophilia B	Disseminated intravascular coagulation	von Willebrand disease	Bernard-Soulier syndrome	b
A patient had repeated episodes of prolonged bleeding following minor trauma. Laboratory tests reveal elevated levels of fibrin degradation products, while normal activated partial thromboplastin time, prothrombin time, thrombin time, and bleeding time. What specific factor deficiency is most likely in this case?	Plasminogen activator inhibitor-1	Activated protein C	Tissue plasminogen activator	Thrombin	a
Which transfusion scenario is likely to cause the most severe transfusion reaction in a patient with no prior transfusion history?	Transfusion A -ve packed cells to AB +ve recipient	Transfusion A -ve packed cells to A +ve recipient	Transfusion of A +ve packed cells to A -ve recipient	Transfusion of A +ve packed cells to O -ve recipient	d
Identify the option that accurately describes the objective of the major crossmatch procedure performed before blood transfusion.	To detect unexpected antibodies in the donor's serum	To detect unexpected antibodies in the recipient's serum	To identify ABO and Rh antigens on donor red blood cells	To identify ABO and Rh antigens on recipient red blood cells	b
Which of the following statements regarding the ABO blood group system is correct?	The anti-A and anti-B antibodies present in plasma are predominantly IgG-type	The ABO system exclusively follows Landsteiner's first law	Blood type O is characterized by the presence of anti-A, anti-B, and anti-H antibodies in plasma	The antigens and antibodies of the ABO system are not fully developed at the time of birth	d
A 28-year-old woman requiring urgent blood transfusion was determined to have an O +ve blood group by routine antisera testing. In an emergency, she was transfused with an O -ve blood transfusion, but she developed a haemolytic reaction. Further laboratory analysis shows she lacks the H antigen on her red blood cells. What is the most appropriate blood type for transfusion?	Packed RBCs of O -ve blood	Packed RBCs of O +ve blood	Only plasma of O -ve blood	Both options a and b are correct	c
A medical student is studying Landsteiner's Law concerning the blood group system. Which of the following statements correctly describes this law?	The plasma of an individual does not contain antibodies against the antigens found on their RBCs	The plasma of an individual does not contain antibodies against the antigens absent from their RBCs	RBCs of an individual do not contain antibodies against the antigens absent in their plasma	RBCs of an individual do not contain antibodies against the antigens present in their plasma	a
Which of the following statements about the Rh blood group is true:	Rh -ve person has anti-D antibodies in the plasma	There are more than 50 Rh antigens	Rh null means the absence of D antigen	Rh blood group has cold agglutinins	b
Considering a scenario where the father's blood type is AB positive, the mother's blood type is A negative, and their child's blood type is O positive. Which of the subsequent statements offers the most accurate conclusion concerning the child's biological parentage?	The couple are the biological parents of this child	Neither the father nor the mother could be the biological parents	Of the couple, only the mother could be the biological parent of this child	Of the couple, only the father could be the biological parent of this child	c
Which of the following parental blood group combinations presents a risk of Rh incompatibility, potentially leading to haemolytic disease in a newborn?	Mother and father are both Rh-negative	Mother and father are both Rh-positive	Father is Rh-negative, and mother is Rh-positive	Father is Rh-positive, and mother is Rh-negative	d
Which of the following is the most appropriate immediate treatment for a newborn having severe haemolytic disease due to Rh incompatibility?	Administer anti-Rh immunoglobulin to the infant	Perform an exchange transfusion using Rh-positive blood	Perform an exchange transfusion using Rh-negative blood	Administer anti-Rh immunoglobulin to the mother	c
A newborn develops jaundice within the first 24 hours of life. The infant's blood group is A positive, while the mother's is O positive. The Direct Coombs test confirms antibody binding on infants' RBCs. Which of the following antibodies is most likely responsible for the positive Coombs test in this infant?	Anti-A, IgG	Anti-A, IgM	Anti-D, IgG	Anti-D, IgM	a
Which of the following best describes the mechanism of anti-D immunoglobulin (RhoGAM)?	Reacts and blocks the Rh antigen on foetal RBCs in the uterus	Suppresses maternal B-cell activation and prevents antibody formation	Directly neutralizes maternal anti-D antibodies	Both a and b	b
A Rh-negative mother delivers a Rh-positive baby. When should RhoGAM be administered to prevent Rh sensitization?	At 12 weeks of gestation and within 24 hours after delivery	At 28 weeks gestation and within 72 hours after delivery	Only if foetal anaemia is detected	RhoGAM is not needed if the first pregnancy is uneventful	b
A 55-year-old man with blood type A +ve presents with acute blood loss after trauma and requires an emergent transfusion. He is mistakenly given O +ve red blood cells. Which immunological mechanism is most likely responsible for an immediate haemolytic reaction in this patient?	IgG-mediated extravascular haemolysis	IgM-mediated complement activation	IgE-mediated anaphylaxis	T-cell mediated cytotoxicity	b
During the storage of packed red blood cells, several metabolic changes occur. Which of the following is a typical storage lesion observed in these units?	Increased 2,3-biphosphoglycerate levels	Decreased pH	Elevated ATP levels	Improved cell deformability	b

## References

[1] (2025). National Medical Commission: competency based undergraduate curriculum for the Indian Medical Graduate Volume I. https://www.nmc.org.in/wp-content/uploads/2020/01/UG-Curriculum-Vol-I.pdf.

[2] Lu C, Xu J, Cao Y (2023). Examining the effects of student-centered flipped classroom in physiology education. BMC Med Educ.

[3] Nichat A, Gajbe U, Bankar NJ, Singh BR, Badge AK (2023). Flipped classrooms in medical education: Improving learning outcomes and engaging students in critical thinking skills. Cureus.

[4] Agarwal M, Sharma P, Goswami A, Mittal R (2024). A 2023 nationwide study on adjustment disorder among first year MBBS students in India. Bioinformation.

[5] Picton A, Greenfield S, Parry J (2022). Why do students struggle in their first year of medical school? A qualitative study of student voices. BMC Med Educ.

[6] Ames S (2023). Work and wellbeing: the dynamism of the eight-hour day tradition. St Mark's Rev.

[7] Singh VK, Tiwari M, Singh S, Kumar S (2024). Faculty perception of scenario-based MCQs, SAQs, and MEQs in medical education at an apex institute. Med Sci Educ.

[8] Brenner JM, Fulton TB, Kruidering M, Bird JB, Willey J, Qua K, Olvet DM (2024). What have we learned about constructed response short-answer questions from students and faculty? A multi-institutional study. Med Teach.

[9] Goyal M, Agarwal M, Goel A (2023). Interactive learning: online audience response system and multiple choice questions improve student participation in lectures. Cureus.

[10] Shrivastava SR, Shrivastava PS (2024). From chaos to clarity: Use of mind maps as a tool to ensure better learning among medical students. Indian J Community Med.

[11] Datta V, Shukla A, Meena JL (2023). Improving the quality of healthcare in resource-constrained settings: is improving undergraduate medical education quality the way out?. BMJ Open Qual.

[12] Ghosh K (2022). Undergraduate medical education in India: need for total modification. J Hematol Allied Sci.

[13] Chandra N, Mehndiratta M, Garg S, Puri D (2018). Current state of medical education in India: a perspective. Indian J Med Biochem.

[14] Hall JE, Hall ME (2020). Guyton and Hall Textbook of Medical Physiology: Third South Asian Edition. https://shop.elsevier.com/books/guyton-and-hall-textbook-of-medical-physiology-3rd-sae/vaz/978-81-312-5773-9.

[15] Gupta DK, Chaudhuri A, Gaine D (2025). A systematic review of self-directed learning in medical education in undergraduate medical students. Curr Med Issu.

[16] Haycocks NG, Hernandez-Moreno J, Bester JC (2024). Assessing the difficulty and long-term retention of factual and conceptual knowledge through multiple-choice questions: a longitudinal study. Adv Med Educ Pract.

[17] Parekh P, Bahadoor V (2024). The utility of multiple-choice assessment in current medical education: a critical review. Cureus.

[18] Choudhari SG, Gaidhane AM, Desai P, Srivastava T, Mishra V, Zahiruddin SQ (2021). Applying visual mapping techniques to promote learning in community-based medical education activities. BMC Med Educ.

[19] Sajadi AS, Babajani A, Maroufi SS, Sarraf N (2024). Using the mind map method in medical education, its advantages and challenges: a systematic review. J Educ Health Promot.

[20] Bradbury NA (2016). Attention span during lectures: 8 seconds, 10 minutes, or more?. Adv Physiol Educ.

